# Abstract of the Proceedings of the Will Co. Medical Society

**Published:** 1861-03

**Authors:** G. S. Thomas, A. W. Heise

**Affiliations:** Secy.; Pres.


### ABSTRACT OF THE PROCEEDINGS OF THE WILL CO. MEDICAL SOCIETY.

The Society met agreeably to order at two o'clock P. M. of Wednesday, Feb. 13th, 1861, in the office of Dr. F. K. Bailey, Joliet. Dr. Bailey President in the chair.

The following gentlemen were elected officers for the ensuing year.

President, Dr. A. W. Heise, Vice President, Dr. E. K. Willard, Secretary, Dr. G. S. Thomas.

Drs. F. K. Bailey, P. B. McKay, and M. J. Johnson, were elected censors. The following are at present members of the Society:

Dr. W. Danforth, Joliet,
Dr. J. R. Casey, Joliet,
Dr. F. K. Bailey, Joliet,
Dr. A. W. Heise, Joliet,
Dr. G. S. Thomas, Joliet,
Dr. E. K. Willard, Wilmington,
Dr. P. B. McKay, Wilmington,
Dr. S. M. Abbott, Wilmington,
Dr. A. W. Bowen, Wilmington,
Dr. O. Corbin, Wilmington,
Prof. A. S. McArthur, (Lind University,) Joliet,
Dr. J. F. Daggett, Lockport,
Dr. H. Folke, Milton,
Dr. M. J. Johnson, Wilmington,
Dr. E. H. Story, Wilmington.

Dr. A. W. Heise read an interesting paper upon Placenta Prævia, which was listened to with much pleasure by all present. Dr. P. B. McKay was appointed to read an Essay before the Society, at the next meeting. On motion, adjourned to meet at one o'clock, Wednesday, P. M., March 13th, at the office of Dr. E. K. Willard, in Wilmington.

G. S. THOMAS, *Secy.*

A. W. HEISE, *Pres.*

